# A Foldable Chip Array for the Continuous Investigation of Seed Germination and the Subsequent Root Development of Seedlings

**DOI:** 10.3390/mi10120884

**Published:** 2019-12-17

**Authors:** Zhao Xi Song, Hui Hui Chai, Feng Chen, Ling Yu, Can Fang

**Affiliations:** 1 Key Laboratory of Luminescent and Real-Time Analytical Chemistry (Southwest University), Ministry of Education, Institute for Clean Energy and Advanced Materials, Faculty of Materials and Energy, Southwest University, Chongqing 400715, China; szx1227@email.swu.edu.cn (Z.X.S.); chh0221@email.swu.edu.cn (H.H.C.); cf19950629@email.swu.edu.cn (F.C.); 2 School of Computer Science and Software Engineering, Southwest University, Chongqing 400715, China

**Keywords:** seed germination, root development, plant cultivation, microfluidic, foldable

## Abstract

Seed germination and seedling root development are important indicators of plant development. This work designed and fabricated a foldable microfluidic chip array for conducting nondestructive and continuous evaluation of seed germination and subsequent seedling development in situ. Each plant chamber has two functional units: seed germination part and root-growth part. The root-growth parts are themselves connected to a single channel designed to provide a uniform culture medium for plant growth. The individual chips are connected into an array using elastic hinges that facilitate the folding and unfolding of the array to accommodate different viewing purposes. In the folded state, the seed germination chambers form a closely spaced array platform to facilitate the comparison of seed germination and plant development characteristics. Unfolding the array facilitates a clear examination of root development within the root-growth parts. The observation window of an individual chip facilitates either the direct examination of the developing seedling (e.g., stems and leaves) or the use of a microscope for examining microscale features (e.g., root tips and root hairs). The potential of the proposed foldable chip array as a new cultivation platform for botanic studies is demonstrated by examining the seed germination and seedling development of tobacco (*Nicotiana tabacum*) under different cultivation conditions.

## 1. Introduction

Seed germination and seedling root development are important indicators of plant development. Germination studies focus on examining the sprouting of seedlings and are widely used for conducting a phenotypic evaluation and evaluating seed quality and variations in seed quality for determining improvement and standardization metrics [1,2,3]. Meanwhile, seedling growth and root system development are major factors affecting plant cultivation and are therefore of particular interest [4,5,6,7]. The distinct development stages associated with germination and seedling development require that conventional germination assay, seedling growth, and root system analysis experiments be conducted using two different experimental settings [8,9]. Seeds in germination experiments are generally placed on germination paper, sand, or a solid agar bed, and incubated for several days. The sprouted seedlings must be transplanted to another container, such as a conical flash or culture tank, with a culture base, such as soil, for further cultivation when conducting seedling growth and root system development analysis. The continuous evaluation of the entire process from seed germination to seedling development in situ would be valuable because seed germination is naturally followed by root and shoot development, which eventually forms an entire plant. Moreover, while the development of shoots and leaves can be evaluated during cultivation, root system development can be examined only at the endpoint of the experiment because the seedlings must be carefully extracted from the culture base and thoroughly washed to observe their structure [5,7]. As such, the nondestructive observation and analysis of root systems in real-time are extremely challenging, and the entire process from seed germination to seedling development cannot be investigated in situ using conventional cultivation methods.

These challenges associated with conventional cultivation methods have been addressed to some extent in the past few years by advances in microfluidics and miniaturized devices with good optical transparency. For example, seed germination, root development, and pollen tubes have all been studied using innovative micro-devices such as polydimethylsiloxane (PDMS)/glass hybrid micro-devices [8,10,11,12,13,14]. Micro-devices have also been developed for conducting novel research previously inaccessible by conventional methods. For example, a novel plant chip array filled with a conventional solid agarose culture medium was developed, where individual seeds could be partitioned from each other, and a number of different agarose conditions could be tested simultaneously using a single plant chip array [12]. A RootChip was developed, where *Arabidopsis* seeds initially germinated and grown in conventional pipettes for several days were transferred into the RootChip for conducting high-throughput plant gene expression analysis [15]. However, these studies have focused either on seed germination or on seedling development, and microfluidic devices have not yet been applied for investigating the entire process from seed germination to seedling development in situ [16,17,18,19,20]. Jiang et al. [21] developed a high-throughput vertical microfluidic chip for analysis of *Arabidopsis* phenotypes. In their study, after *Arabidopsis* seeds were trapped in the culture channel, the top part of the channel above the seed holding sites was manually cut off by a razor blade. The level of growth medium in the device was adjusted by slowly flowing a growth medium into the device. Manual cutting may introduce variation between chips.

The present study developed a foldable microfluidic chip array to facilitate the continuous observation and comparison of seed germination and consequent seedling development. The observation window of an individual microfluidic chip facilitates either the direct examination of the developing seedling (e.g., stems and leaves) or the use of a microscope for examining microscale features (e.g., root tips and root hairs). The potential of the proposed foldable chip array as a new cultivation platform for botanic studies is demonstrated by examining the seed germination and seedling development of tobacco (*Nicotiana tabacum*) under different cultivation conditions. The germination rate and growth potential of the seeds are characterized, and the microstructures of the root systems (root tips) are examined nondestructively.

## 2. Materials and Methods

### 2.1. Materials and Reagents

We purchased *N. tabacum* seeds from Shangong (Shanghai, China). PDMS elastomer kits (Sylgard 184) were obtained from Dow Corning (Midland, TX, USA), and blue food color dye was purchased from Dongguan Tianzhi Cai Food Factory (Dongguan, China). All other chemicals were purchased from Aladdin Chemical Reagent Co., Ltd. (Shanghai, China). Chemicals were used as received and prepared in ultrapure water (PURELAB flex system, ELGA Corporation, Paris, France) without further purification.

### 2.2. Design and Fabrication of Foldable Plant Array Chip

**Figure 1:**
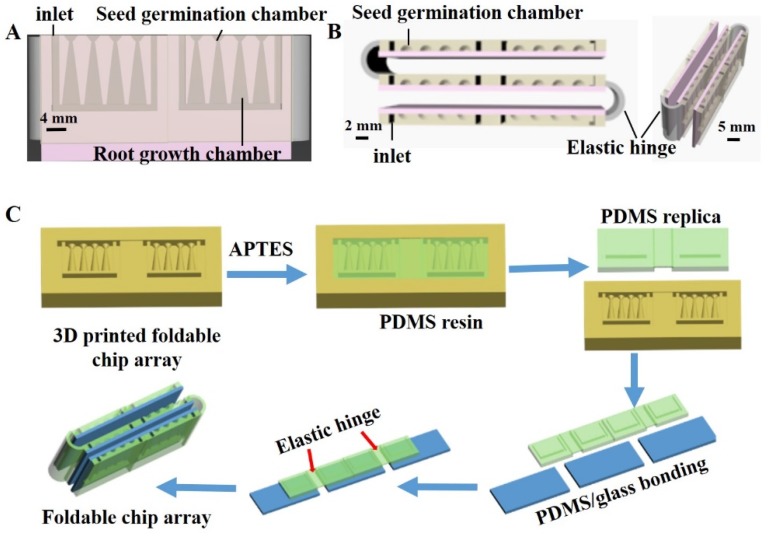
Design and fabrication of foldable microfluidic chip array: (**A**) front view and (**B**) top view; (**C**) individual chip fabrication and application of elastic hinges for achieving an array structure.

The design of the foldable microfluidic chip array is illustrated in Figure 1. Each chip in the array is composed of a structured PDMS component and a glass slide, where the PDMS component is designed to include four seed germination ports with connected root-growth ports that are themselves connected to a channel designed to provide a uniform culture medium for plant growth. The interface between a seed germination port and its connected root-growth port forms a neck-like structure that was designed to permit part of the seed to make direct contact with the culture medium while preventing it from falling in the root-growth port. The diameter of the neck is therefore adjusted according to the size of the seeds under study. Here, the neck diameter employed was 0.4 mm to accommodate an *N. tabacum* seed, which has a diameter of around 0.42 mm. The root-growth portion was designed to provide sufficient space for root system development. The chambers and liquid flow channel were formed by bonding the structured PDMS component with a glass slide, while the open liquid inlets were used for culture medium solution supply and removal. The width and depth of the channel are 1 mm and 13 mm, respectively. In addition, different reagents can be introduced into the growth chambers for setting different cultivation conditions. The individual chips are connected into an array using elastic hinges that facilitate the folding and unfolding of the array for different viewing purposes. When the elastic hinge is folded, the seed germination chambers form an array platform to facilitate the comparison of seed germination and plant development characteristics. Unfolding the array facilitates a clear examination of root development within the root-growth portion by either visual inspection, a Root Analysis System, or a microscope.

The fabrication of the foldable microfluidic chip array is illustrated in Figure 1C. The structured PDMS was fabricated using a standard PDMS-based replica-molding technique. First, the mold for the PDMS replication was printed using a MoonRay three-dimensional (3D) printer. The 3D-printed mold was treated by air plasma (Agilent Technologies Inc., Palo Alto, CA, USA) for 5 min and then salinized by emersion in a 0.1% (3-aminopropyl) triethoxy silane (APTES) absolute ethanol solution for 2 h. The APTES-treated mold was washed in 95% ethanol, blow-dried with nitrogen, and then dried in vacuum at 75 °C for 2 h. The PDMS replica was generated from the mold by mixing PDMS and a curing reagent at a mass ratio of 1:10, casting in the mold, and then curing at 60 °C for 2 h. Finally, the structured PDMS and glass slides were subjected to plasma treatment for 4 min and firmly bonded to form the PDMS/glass hybrid chip.

### 2.3. On-Chip Seed Germination and Plant Cultivation

First, each chip was completely filled with a 75% alcohol solution for 2 h for sterilization, and the chip was drained and allowed to dry in air. Then, each chip was filled with 115 μL of Hoagland’s culture solution. Then, a single *N. tabacum* seed was placed in each of the individual seed germination chambers. The assembled chip array was then covered using a transparent acrylic jar (diameter = 90 mm, height = 150 mm) to create a closed environment for plant growth while reducing the evaporation of water. The plants were cultivated in an artificial climate incubator (LAC-250HPY-2, Shanghai Long Yue Instrument Inc., Shanghai, China). The standard culture conditions of the incubator, unless otherwise stated, were as follows: temperature, 26 °C during the day and 24 °C at night; relative humidity, 80%; light for 16 h during the day with 8 h of darkness. The culture solution was refilled every 12 h, but the seeds and seedlings remained untouched throughout the experiment to avoid damage to the seedlings. The seed germination and seedling development processes within the foldable chip arrays were characterized as follows.

#### 2.3.1. Seed Germination

The seeds were imaged by a camera and examined every 24 h for signs of germination. The number of germinated seeds *N_G_* after 2 days was counted, and the ratio of *N_G_* to the total number of seeds tested *N_T_* was adopted as the germination percentage (GP) as follows:

GP = *N_G_/N_T_* ×100% (1)

#### 2.3.2. Seedling Growth

Germinated *N. tabacum* seeds were further monitored to investigate their shoot and root development. Briefly, the lengths of the shoots and leaves were directly measured daily as they grew above the germination wells. The root-growth potential *G_p_* was also determined as follows:

*G_p_* = (*L_n_* − *L_n−1_*) (2)

where *L_n_* is the root length on the nth day, and *L_n−1_* is the root length 1 day prior. 

In addition, a Root Analysis System (WinRHIZO, Regent Instruments, Ottawa, ON, Canada) was used to examine the roots. Because of the good optical transparency of the PDMS/glass hybrid device, plants grown in the chip can be directly placed into the scanner and measured on different cultivation days. The scanner can automatically analyze several root parameters, such as the root length, root number, and root surface area. Moreover, the root microstructures of the seedlings, such as the root hairs and root tips, were examined through a microscope.

For comparison, we also applied conventional seed germination and seedling analysis methods for of *N. tabacum*. Briefly, 100 *N. tabacum* seeds were evenly arranged on an agar bed in a 100 mm diameter glass Petri dish, and the GP was calculated as described above. Germinated *N. tabacum* seeds were then placed in a conventional conical flask with an agar-based medium and cultivated in the artificial climate incubator under standard conditions. As before, the lengths of the shoots and stems were directly measured daily, and their root systems were carefully extracted from the conical flask at the endpoint of the cultivation period for examination.

### 2.4. On-Chip Screening the Effects of Salt and Drought Stress on Seed Germination and Plant Cultivation 

Finally, the proposed chip array was applied for evaluating the effects of salt and drought stresses on *N. tabacum* seed germination and root development to provide a more practical demonstration of the benefits of the proposed chip array. Briefly, the culture medium with an NaCl concentration of 150 mM was added into the channel to impose salt stress, while the culture media with 10% (w/w%) PEG6000 was added into the channel to impose drought stress, and the seed germination and consequent root development were evaluated as described above.

## 3. Results

### 3.1. Characterization of the Foldable Plant Chip Array

**Figure 2:**
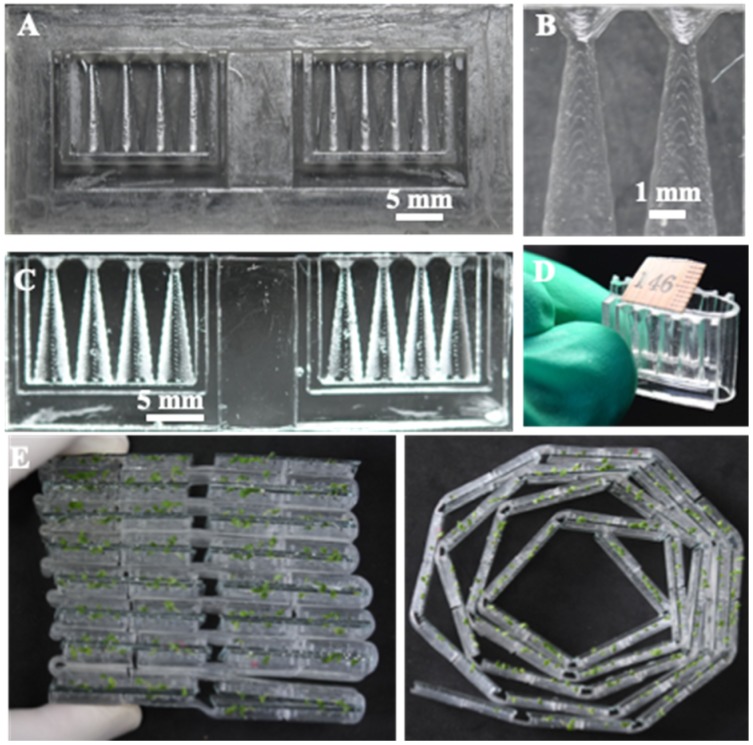
A 3D-printed mold and its corresponding PDMS replica for the prototype array employed in the present study: (**A**) a 3D-printed mold; (**B**) half cone structure for clamping *N. tabacum* seeds; (**C**) corresponding PDMS replica of the 3D-printed mold; (**D**) array folding owing to the elastic PDMS hinge; (**E**) multi-stacked foldable chips.

The 3D-printed mold and its corresponding PDMS replica for the prototype array employed in the present study are shown in Figure 2. A half conical shape seed germination well and root-growth portion can be clearly observed. The “neck” structure prevents the falling of the seed into the root-growth chamber and the elastic hinge enables folding of the chip, as shown in Figure 2D. The elastic hinge structure also provided a potential in high-throughput screening because single chips can be assembled into an array format. In addition, the elastic hinge also provides flexibility in the way to assemble the array. As shown in Figure 2E, multi-chips can be stacked to facilitate the observation and comparison between chambers.

**Figure 3:**
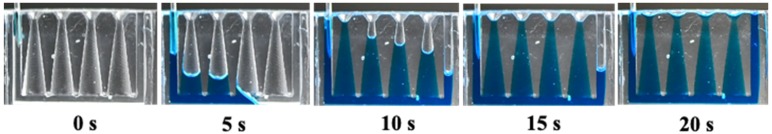
Growth medium transport characterization in a single microfluidic chip: chip filled with culture medium; and after adding blue food dye to the medium through the liquid input.

We evaluated the transportation of growth medium on microfluidic chips. The blue food dye was added into the culture medium through the liquid input, and changes in the color of the solution in the liquid transport channel and the germination and growth chambers were then evaluated every 5 s via image capture. As shown in Figure 3, the solution gradually filled the root-growth portions and the outlet channel. Once the outlet channel fulfilled with the solution, the injection from the inlet was stopped. The total solution volume is 115 µL. The ‘neck’ between the germination well and root-growth portion prevented the leaking of solution from the germination well. 

### 3.2. Seed Germination and Plant Cultivation on a Foldable Chip Array

**Figure 4:**
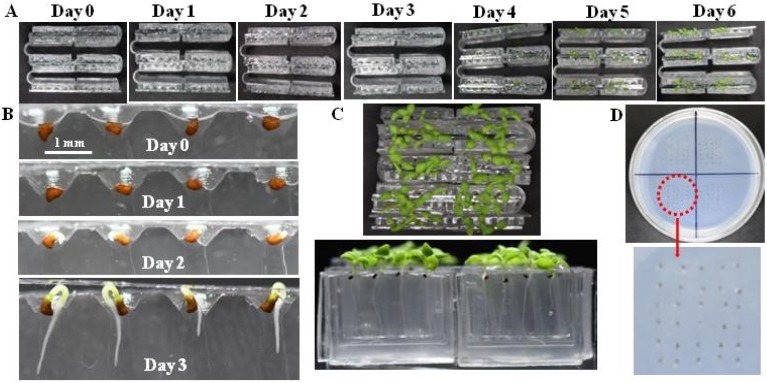
On-chip seed germination testing: (**A**) images of seed germination and seedling growth over successive days; (**B**) images of seed germination chambers from day 0 to day 3; (**C**) different views of seedlings after cultivation for 10 days; and (**D**) conventional agar bed seed germination method.

The results of on-chip seed germination and seedling growth testing are shown in Figure 4A–C, while the conventional agar bed seed germination method is shown in Figure 4D. The images of seed germination and seedling growth at different times shown in Figure 4B and the different views of seedlings after cultivation for 10 days shown in Figure 4C demonstrate the benefits of the proposed chip arrays. Meanwhile, the images of seed germination chambers from day 0 to day 3 shown in Figure 4B demonstrate that the seeds within the germination chambers can be compared easily. The GP obtained for 100 *N. tabacum* seeds on the foldable chip array was 98.2%, which is very similar to the GP obtained for the conventional agar plat-based test (98.5%). In addition, we note that the proposed chip array makes the observation and imaging of sprouting seeds highly convenient, and the primary root emerging from the seed can be very clearly observed and characterized.

**Figure 5:**
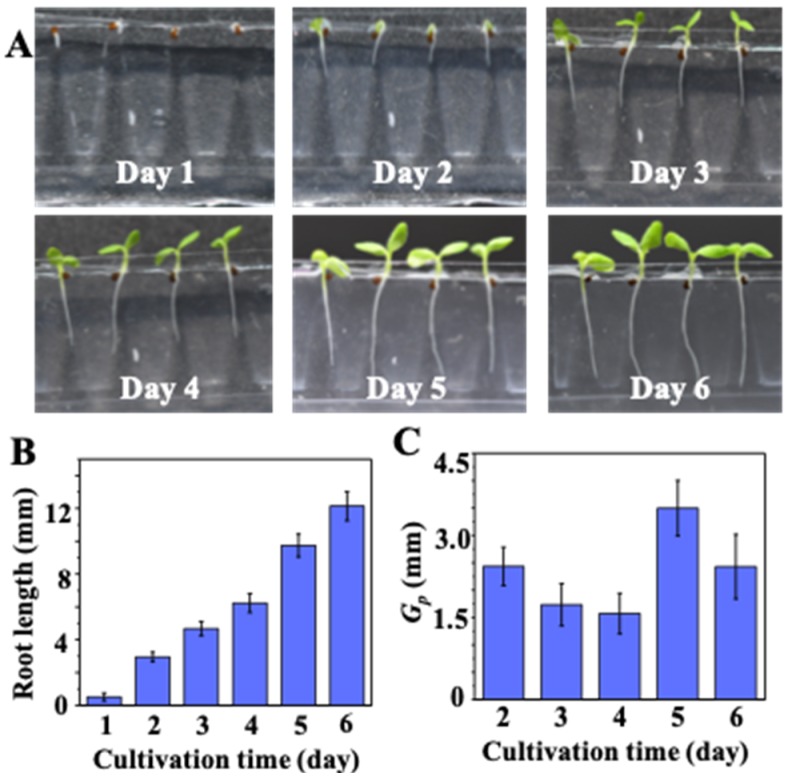
Nondestructive examination of on-chip seedling growth: (**A**) on-chip seedling growth over a period of days; (**B**) on-chip measured average root lengths on successive days (n = 20); (**C**) corresponding root-growth potentials (*G_p_*) of on-chip-cultivated seedlings (n = 20).

In conventional plant cultivation experiments, the germinated seeds on the agar, paper, or towel bed have to be transferred into another culture container, such as conical flasks. In this study, the growth of germinated seeds was continuously examined without the need for transfer. The nondestructive examination of on-chip seedling growth is illustrated in Figure 5A. Both shoots and roots of the developing seedlings can be clearly observed daily. In addition, nondestructive observation of root-growth allows the seedling growth kinetics to be determined directly. As shown in Figure 5B, the average root length increased to 6 ± 2 mm, 8 ± 2 mm, and 12 ± 2 mm after 4, 5, and 6 days of cultivation, respectively. The calculated *G_p_* thus is 1.9 ± 0.2 mm, 1.6 ± 0.22 mm, 1.5 ± 0.2 mm, and 3.5 ± 0.2 mm at day 2, 3, 4, and 5, respectively. The values of *G_p_* shown in Figure 5C indicates that the most rapid development of the *N. tabacum* seedlings was on day 5. 

**Figure 6:**
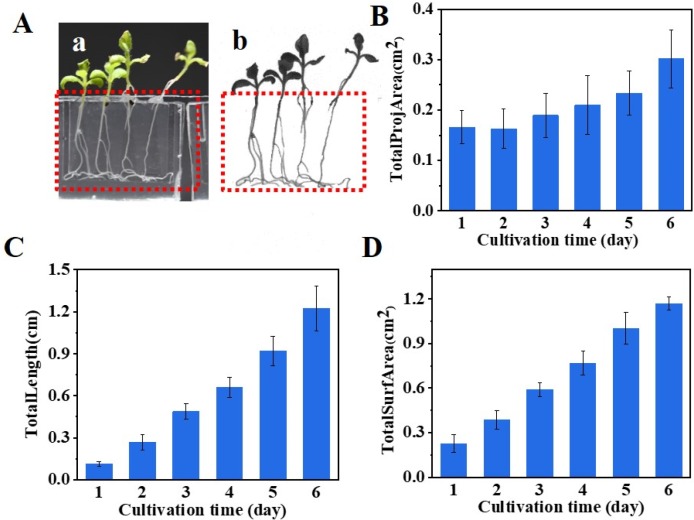
Nondestructive examination of seedling growth on-chip (**A**) extraction of root information by using a WinRHIZO root scanner. (**a**) Camera picture. (**b**) Plant image extracted from the root scanner: growth kinetics of total root projection area (**B**), total root length (**C**), and total root surface area (**D**) characterized by the scanner (n = 20).

The flexibility given by the elastic hinge enables the transparent chip to be directly scanned by the WinRHIZO scanner. As images shown in Figure 6A, roots in the root-growth portion can be clearly observed. On-chip observation not only avoids the destruction of the roots, but also allows for the tracking of the development of the same plant at different time points, which is a key parameter in quantifying the growth rate and growth potential of a seedling. The changes of total projection area (Figure 6B), total root length (Figure 6C), and total surface area (Figure 6D) measured by the WinRHIZO scanner depict the growth kinetics of the *N. tabacum* seedlings.

**Figure 7:**
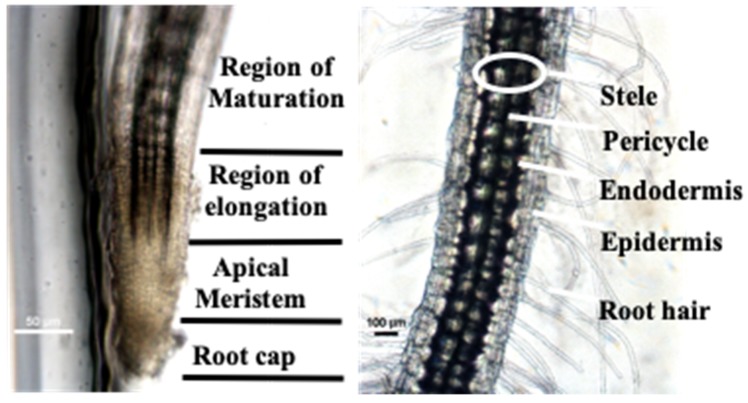
Nondestructive observation of root microstructures through a microscope.

Moreover, the root hair, meristematic zone of plants, and cells can be directly observed through a microscope without fluorescent staining because of the excellent optical transparency of the PDMS/glass hybrid chip (Figure 7). The root systems of seedlings cultured in a conventional conical flask can only be examined at the endpoint of the experiment because the roots must be extracted from the culture base. This demonstrates the benefits of the proposed microfluidic chip for facilitating the continuous examination of seed germination and seedling development processes in situ, which differs markedly from conventional experimental germination assay and seedling development evaluation methods and previously reported germination arrays and root chips that tend to focus on either germination assay or seedling development, but not both [22,23].

### 3.3. Analysis of the Effects of Salt and Drought Stresses on Seed Germination and Seedling Development on-Chip

**Figure 8:**
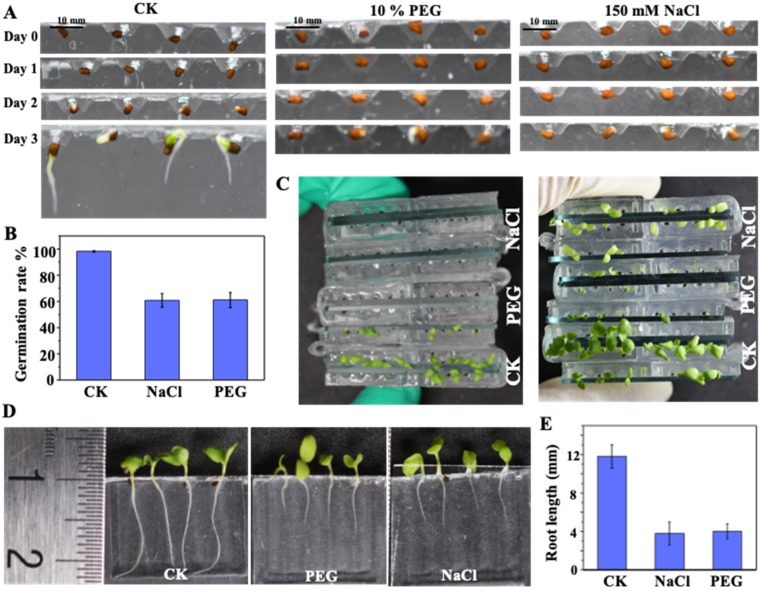
Seed germination and seedling development under salt stress (150 mM NaCl), drought stress (10% PEG), and standard (CK) conditions: (**A**) seed germination under salt, drought, and CK conditions; (**B**) on-chip germination rate under the different growth conditions; (**C**) and (**D**) seedling development corresponding to the different growth conditions in (**A**); and (**E**) average root lengths under the different growth conditions.

Seed germination and seedling development results obtained for *N. tabacum* using the proposed chip array under salt stress (150 mM NaCl), drought stress (10% PEG), and standard (CK) conditions are illustrated in Figure 8. The images in Figure 8A demonstrate that the use of the microfluidic chip makes the effects of environmental stresses on seed germination clearly observable, where most of the seeds germinated under the CK condition, while the NaCl and PEG conditions both inhibited seed germination. As shown in Figure 8B, the corresponding GP under CK, NaCl and PEG conditions are 98.2 ± 0.5%, 60.25 ± 0.5%, and 60.51 ± 0.5%, respectively. In addition, the practical benefit of using the chip array for comparing seedling growth under the various environmental conditions is illustrated in Figure 8C. The corresponding effects of the environmental conditions on seedling development are also clearly exhibited in Figure 8D, where the environmental stress conditions are shown to slow seedling development relative to the CK condition. Finally, the nondestructive and continuous observation of seed germination and root development in the proposed chips allowed for the seed germination and seedling growth kinetics to be directly determined, as shown in Figure 8E, which thereby provides quantitative analysis corresponding to the qualitative analyses provided in Figure 8D.

**Figure 9:**
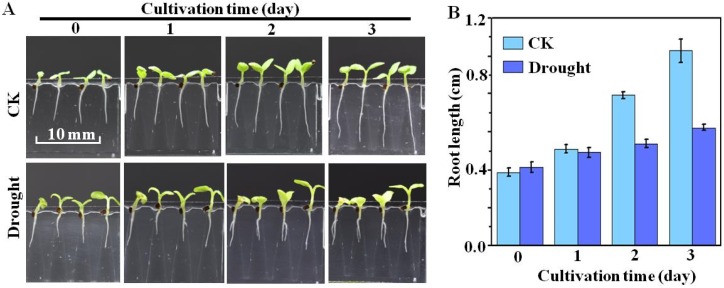
Results of seed germination conducted under the CK condition with subsequent seedling development continued for 3 days under either the standard (CK) or drought (10% PEG) condition: (**A**) images of seedling development after successive days; and (**B**) average root lengths obtained after successive days (n = 20).

The microfluidic chip design also allows for the environmental conditions of the growth medium to be varied in situ during the uninterrupted seed germination and seedling development processes. This is demonstrated in Figure 9, which presents the results of seed germination conducted under the CK condition with subsequent seedling development continued for 3 days under either the CK or PEG condition. The deviations between seedling development under the two conditions over the three-day period are both qualitatively and quantitatively apparent. As shown in Figure 9B, the root length increased to 9 ± 2 mm, and 10 ± 2 mm after two and three days of cultivation under CK, compared to 5 ± 1 mm and 6 ± 1 mm under drought.

## 4. Conclusions

This study proposed a foldable microfluidic chip array for the continuous monitoring of seed germination and seedling development in situ. In the folded state, the seed germination chambers form a closely spaced array platform to facilitate a high-throughput comparison of seed germination and plant development characteristics. Unfolding the array facilitates a precise examination of root development within the root-growth chambers. Moreover, the root hair, meristematic zone of plants, and cells can be directly observed through a microscope without fluorescent staining because of the excellent optical transparency of the PDMS/glass hybrid chip. As such, the proposed microfluidic cultivation system provides a new analytical tool for botanical research that is generally applicable to a number of purposes such as germination testing, stimuli screening, and root development experiments.
